# Editorial: Next research perspectives in pediatric cardiology for imaging and interventions

**DOI:** 10.3389/fped.2022.986222

**Published:** 2022-08-18

**Authors:** Olivier Villemain, Alvise Guariento, Zakaria Jalal

**Affiliations:** ^1^Division of Cardiology, Department of Pediatrics, The Hospital for Sick Children, University of Toronto, Toronto, ON, Canada; ^2^Pediatric and Congenital Cardiac Surgery Unit, Department of Cardiac, Thoracic and Vascular Sciences and Public Health, University of Padua, Padua, Italy; ^3^Department of Pediatric and Congenital Cardiology, M3C National Reference Centre, Bordeaux University Hospital, Bordeaux, France

**Keywords:** pediatrics, cardiology, congenital heart diseases (CHD), technology, innovation

Pediatric and congenital cardiology has developed its own challenges. Indeed, the management of these patients has evolved considerably over the last 20 years and new solutions can be proposed to adapt to this. To do so, we must capitalize on emerging imaging technologies to improve our diagnostic capabilities, on the advancement of artificial intelligence applied to precision medicine, as well as on a better use of the means available to the physician to manage his patients.

In this Research Topic (*Next Research Perspectives in Pediatric Cardiology for Imaging and Interventions*), Avesani et al. remind the importance of 3D echocardiography and multimodality imaging in the assistance of percutaneous interventions in congenital heart diseases (CHD). Evoking the risks of X-ray exposures, the authors present in this state-of-the-art review all the new imaging techniques available in the cath lab. They define the role of these techniques and their potential to guide the intervention in real time. Through the description of specific application examples, such as shunt closures, valve interventions, or fetal cardiac interventions, the reader of this article will be able to understand how volumetric ultrasound imaging (3D echocardiography) can bring added value to certain procedures. Moreover, 3D echocardiography can now be “merged” in real time with other imaging techniques (angiography or CT). Beyond the technological interest and the curiosity that this brings, these tools must now find their indication for the management of our patients.

In another article in this Research Topic, Venet et al. proposes a review of the role of nuclear imaging for CHD management. This imaging technique requires a very specific technical platform and expertise which are described by the authors. Listing the multiple possible clinical applications, summarized in their Figure 3, Venet et al. also recall the risks, the limits, and the specificities of nuclear imaging in pediatrics. Through this review paper, the authors will help their readers to better understand why this imaging technique should be integrated into the management of certain situations in CHD such as inflammation/infection assessment, shunt quantification, lung perfusion, or heart tumors detection (see Figure 3 of the paper as an overview of potential indications).

After reading these two publications on imaging techniques, it seems obvious that the analysis and management of complex data in pediatric and congenital cardiology will be a major challenge in the coming years. But beyond the imaging data, it is our ability to integrate all the information at our disposal that will allow us a better personalized approach of the medical practice. Van den Eynde et al. develops this perspective by integrating Medicine-Based Evidence (MBE) in the future of CHD management. In this approach, big data and deep learning techniques are implemented to interrogate treatment responses among patients in real-world clinical practice. The authors summarize the limits of evidence-based medicine (EBM) and demonstrate that the management of our large masses of data should allow us to provide new solutions.

But beyond the technical or technological developments, optimization of the daily management of a patient with CHD is a major issue. Arbic et al. discuss the thorny issue of the organization of an echocardiography laboratory and the sonographer's role. Recalling the heterogeneity of these echo lab organizations worldwide, the authors seek to understand the advantages and limitations of each system (with or without sonographer). Describing the impact of these systems, whether clinical, academic, or financial, the authors' goal is not to determine which system would be the most efficient but to help their readers determine which organization would best suit their center, their patients, and their needs.

Bottom line, this Research Topic published in Frontiers in Pediatrics provides a fresh look at some of the major topics in pediatric and congenital cardiology. We hope that our readers will enjoy discovering it.

## Author contributions

OV wrote the first draft of the manuscript. All authors contributed to manuscript revision, read, and approved the submitted version.

## Conflict of interest

The authors declare that the research was conducted in the absence of any commercial or financial relationships that could be construed as a potential conflict of interest.

## Publisher's note

All claims expressed in this article are solely those of the authors and do not necessarily represent those of their affiliated organizations, or those of the publisher, the editors and the reviewers. Any product that may be evaluated in this article, or claim that may be made by its manufacturer, is not guaranteed or endorsed by the publisher.

